# Case Report: Persistency Pneumococcal Polysaccharide in Cerebrospinal Fluid During a Post Pneumococcal Chronic Aseptic Meningitis: Coincidental or (Auto-)Inflammatory Embers

**DOI:** 10.3389/fped.2022.762457

**Published:** 2022-02-09

**Authors:** Manon Ladeveze, Yann Dumont, G. Boursier, Frederic Batteux, Perrine Mahe, Aurelie Bensimon Borrull, Guillaume Sarrabay, Karine Bollore, Edouard Tuaillon, Sylvain Godreuil, Eric Jeziorski

**Affiliations:** ^1^Département Urgences Post-Urgences, CEREMAIA, CHU de Montpellier, Montpellier, France; ^2^Laboratoire de Bactériologie, CHU de Montpellier, Montpellier, France; ^3^Laboratoire de Génétique des Maladies Rares et Autoinflammatoires, Département de Génétique Médicale, Maladies Rares et Médecine Personnalisée, CEREMAIA, CHU de Montpellier, Univ Montpellier, Montpellier, France; ^4^Plateforme d'ImmunoMonitoring Vaccinal (PIMV), Laboratoire d'Immunologie, Hôpital Cochin, Paris, France; ^5^Plateforme Exploration de cellules rares en immunologie et infectiologie, CHU de Montpellier, Montpellier, France; ^6^PCCEI, INSERM, Univ Montpellier, Montpellier, France; ^7^Laboratoire de virologie, CHU de Montpellier, Montpellier, France; ^8^UMR IRD224-CNRS5290-UM MIVEGEC, Univ Montpellier, Montpellier, France

**Keywords:** pneumococcal meningitis, pneumococcal cell wall components, aseptic meningitis, chronic aseptic meningitis, innate immunity

## Abstract

We report the case of a 9-months-old boy that has presented a steroid-dependent post-pneumococcal chronic aseptic meningitis was associated with persistence of pneumococcal cell wall components in cerebrospinal fluid during more than 20 months. Suggesting that this antigenic persistence could be involved in post-infectious manifestations through innate immunity response.

## Introduction

Pneumococcal meningitis is a potentially life-threatening infection which often cause sequelae despite antimicrobial chemotherapy. Corticosteroids are indicated to reduce sequelae (1). In this context, steroids-dependent post-pneumococcal chronic aseptic meningitis is a rare complication that have been described (2) for which pathophysiology is not clearly understood. The following is the description of a child that presents chronic aseptic meningitis following acute pneumococcal meningitis associated with persistence of pneumococcal cell wall components in cerebrospinal fluid during more than 20 months controlled by prolonged steroids therapy.

## Case Report

A previously healthy 9-months-old boy was admitted on December 2017 to the pediatric intensive care unit of Arnaud de Villeneuve Hospital (Montpellier, France) for febrile meningeal syndrome. In his history, he received two doses of combined vaccine (diphtheria, tetanus, acellular pertussis, inactivated poliovirus, hepatitis B, *Haemophilus influenzae* type b) and two injections of 13-valent pneumococcal conjugated vaccine.

At hospital admission, the CRP level was 543 mg/liter. Cerebrospinal fluid (CSF) samples contained 28WBC/mm^3^ (including 50% granulocytes), 1.65 g of protein/L, and <0.1 mmol of glucose/L. Gram staining revealed gram-positive diplococci. Empirical therapy consisting of intravenous cefotaxime (300 mg/kg/day) and dexamethasone (0.15 mg/kg/6h) was started. CSF and blood culture were positive to *Streptococcus pneumoniae* (SP) serotype 24F.

At day one a status epilepticus occurred, rapidly stopped with antiepileptic therapy. Cerebral Magnetic Resonance Imaging (MRI) showed osteitis foci in both mastoids without empyema vasculitis or ischemia. Apyrexia was quickly obtained, and dexamethasone stopped after 4 days.

On the fifth day, meningeal syndrome reappeared. CRP rebounded (347 mg/liter), CSF culture was sterile. Brain MRI revealed a lepto-pachymeningitis. Dexamethasone was reintroduced with a progressive decrease.

On the tenth day, antibiotic therapy was switched for amoxicillin and rifamicin for a total duration of 21 days. On the fourteenth day, with dexamethasone 0.15 mg/kg/day, fever reappeared. CSF culture was sterile. Symptomatology regressed with dexamethasone increase. Dexamethasone was switched for prednisolone 3 mg/kg/day, on the twenty-second day.

**Figure 1:**
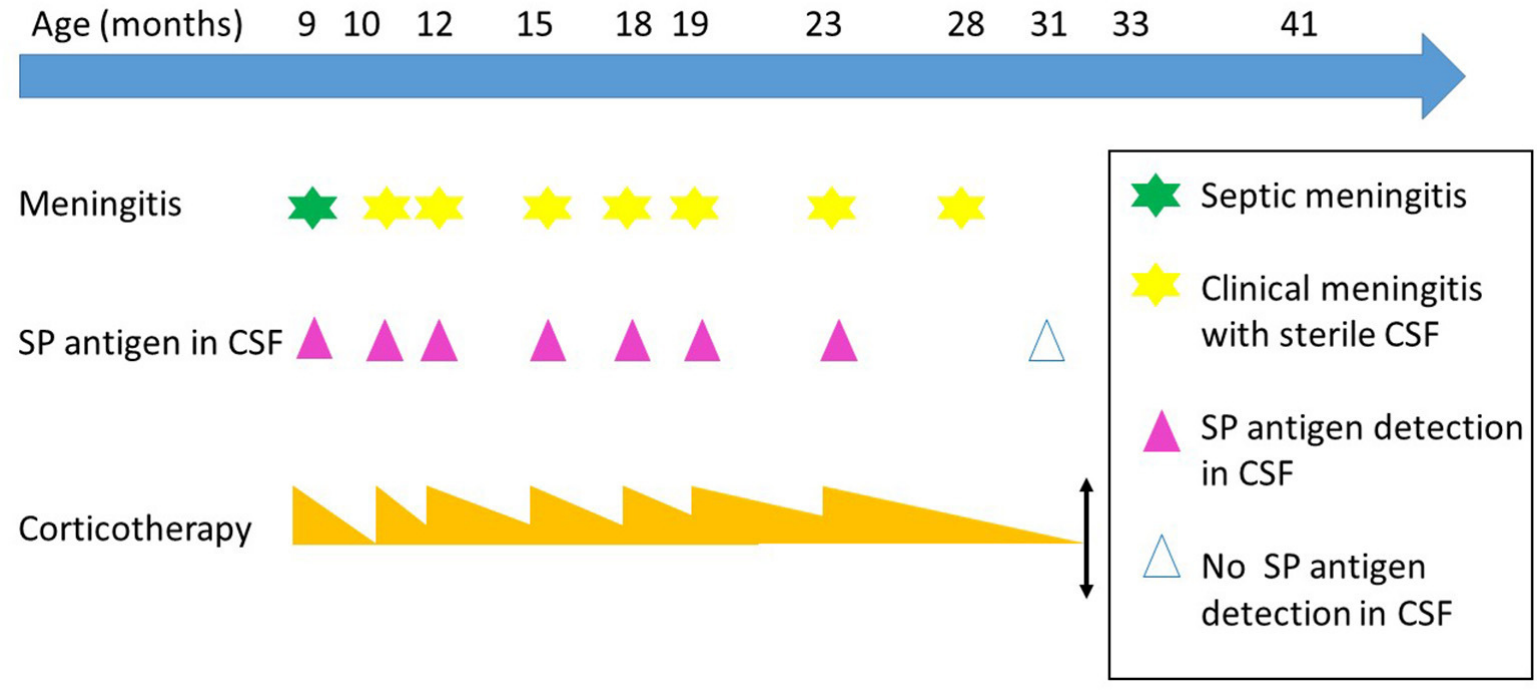
Meningitis episodes over the times. SP, *Streptococcus pneumonia;* CSF, cerebrospinal fluid. Evolution over the time, the steroids therapy is represented by orange triangle, the height is proportional to the dosage of the medication.

Thereafter despite a very gradual decrease, aseptic meningitis (AM) reappeared six times daily dose of prednisolone was reduced under 10 mg (episode at 10, 15, 18, 19, 23 and 28-month-old). Every time, CRP risen again over 60 mg/L, CSF showed an increase of WBC (predominance of granulocytes), moderate elevation of CSF protein concentration, whereas CSF tested negative for SP by PCR and microbiological culture. The Sofia SP fluorescence immunoassay (FIA; Quidel) (SPFI) revealed persistent positive SP soluble antigens in CSF. Viral searches were negative, excepted at 19-month-old where enterovirus was weakly positive. Every time the symptomatology was quickly normalized increasing prednisolone. Away from an AM episode, CSF was normal except persistence of SP antigen (Figure 1).

When he was 31-months-old, during a common fever without meningitis manifestation, SP antigen in CSF was negative. Prednisolone therapy was definitively stopped at 33-months-old. Brain MRI control was normal. Signs of meningeal irritation did not reoccur during 8 months of follow-up. At a distance neurodevelopment was perfect.

Immunological explorations were performed: Howell-Jolly bodies were absent. Immunoglobulin levels, post-vaccinal antibody titers (Tetanus, Poliomyelitis, Haemophilus influenza type b), complement's fractions, lymphocyte subpopulations and extended lymphocyte phenotyping were normal. Deep exploration of the complement was normal (especially no inhibitory C1 deficiency). To avoid an auto-immune disease we have check for antinuclear antibodies and anti-neutrophil cytoplasmic antibody that were absent. HIV serology and search of chronic septic granulomatosis were negative. Trio whole exome sequencing was performed for the proband and his unaffected parents to explore all known genes involved in immunodeficiencies and auto-inflammatory syndromes, none pathologic mutation was observed in these genes (Supplementary Data 1). Cerebro-spinal MRI eliminated a dermoid cyst.

Dosage of cytokines in CSF were performed during the AM of the 19th-month and away from any AM: IL1 was stable while IL6 and IL8 clearly increased in AM CSF.

In order to evaluate specific immunological response, we perform antibodies dosage against pneumococcal capsular polysaccharides. Regarding the serotype 24F, specific ELISA and opsophagocytic assay revealed the production of specific antibodies but non-functional (Table 1). Written informed consent was obtained from the legal guardian for the publication of any potentially identifiable images or data included in this article.

**Table 1:** Specific evaluation of antibodies against pneumococcal capsular polysaccharide in serum.

***Streptococcus pneumoniae* serotype**	**4**	**6B**	**9V**	**14**	**18C**	**19F**	**23F**	**24F**
Antibody level at 20 months	0.26 mg/L	**1.28 mg/L**	0.77 mg/L	**11.7 mg/L**	0.55 mg/L	**1.85 mg/L**	0.51 mg/L	**1.47 AU/L**
Antibody level at 30 months	0.15 mg/L	**1.93 mg/L**	0.98 mg/L	**4.6 mg/L**	**1.01 mg/L**	**1.12 mg/L**	**1.01 mg/L**	0.3 AU/L
OPA test at 20 months	−	−	−	+	−	−	−	−

*Immunogenicity was primarly by measuring anticapsular polysaccharide antibody levels using ELISA. For serotype 4, 6B, 9V, 14, 18C, 19F, 23F antibody level of 0.35 mg/L is considered as protective. For serotype 24F, in the absence of standardized dosage, Arbitrary Unit (AU/L) were used, antibody level of 0.56 AU/L is considered as protective*.

*Functional antibody activity was measured using opsophagocytic assay (OPA)*.

*Result in bold are considered as positive*.

## Discussion

Brouwer et al. (1) reported two cases of aseptic meningitis after pneumococcal meningitis. With persistence of pneumococcal cell wall components in CSF was proven by after pneumococcal meningitis. Using a rapid test (the Binax NOW™ SP antigen test, Abbott), in both cases, a persistence of C polysaccharide antigen (CPA) in CSF was showed. In this case report we used a lateral-flow immunoassay with a fluorescence analyzer to detect Streptococcus pneumonia cell wall polysaccharide in CSF.

Pneumococci have a unique structure that includes a capsule, a polysaccharide cell wall and a cell membrane. C polysaccharide cell wall antigen is common to all *Streptococcus pneumoniae* serotypes. It should be noted that *Streptococcus mitis* and *Streptococcus oralis* are also known to harbor pneumococal C polysaccharide like antigens, however they are uncommun cause of bacterial meningitis.

Although the pathophysiological phenomenon remained unknown, the authors hypothesized the presence of inflammatory phenomena in response to the persistence of pneumococcal cell wall components in CSF. In addition, in the two reported cases, patients had responded to an anti-inflammatory treatment (salicylic acid and prednisolone, respectively).

In our patient, we relate the occurrence of repeated AM episodes with corticosteroid dependence in the aftermath of SP meningitis. This strongly suggests a chronic meningeal inflammation contained by corticosteroid therapy.

Using the SPFI we observed the antigenic persistence of CPA in CSF during more than 20 months. To our knowledge it is the longest duration of detection of pneumococcal soluble antigen (Angoulvant et al. reported a duration of 90 days) (2, 3).

In our observation, relapsing meningitis episodes have stopped after negativisation of SPFI suggesting the persistence of CPA could participate in the pathophysiology of aseptic meningitis. A variety of pneumococcal cell wall compounds are known to be proinflammatory. This was showed after experimental approach as intracisternal inoculation of heat-killed unencapsulated pneumococci, purified pneumococcal cell wall, cell wall lipoteichoic acid, or cell wall peptidoglycan (4). A clinical study has shown that persistence of pneumococcal compounds such as pneumolysin, that doesn't belong to the cell, wall has even been associated with mortality (5).

In our case, antibiotics have killed the germs but the elimination of bacterial compounds from the CSF seems to have been faulty. We then hypothesized that in the sanctuary space of CSF, recognition of different bacterial pathogen-associated molecular patterns (PAMPs) by antigen presenting cell (APCs) has activated an immune reaction. Thus, as mechanisms leading to aseptic meningitis, we can properly suspect a persistent inflammatory response of the host, triggered by a direct interaction of the pneumococcal cell wall components with host's TLRs, which maintain the production of pro-inflammatory cytokines related to innate immunity.

By performing cytokine assays in the CSF away and during a meningeal episode, we logically observed an increase in the level of IL6 and IL8 that are pro-inflammatory cytokines, involved particularly in innate immunity and auto-inflammatory pathways. Unfortunately, cytokine assays during meningeal inflammation were carried out on the 19th month episode (CSF weakly positive for enterovirus). Thus, we cannot distinguish phenomena related to the lifting of immunosuppression and what could be due to the potential immune stimulation induced by enterovirus (this episode occurred concomitantly with a corticosteroid therapy decrease). In fact, the meningeal syndrome resolved immediately after increasing prednisolone therapy.

Experimental animal models have shown that prognostic of bacterial meningitis is related to the severity of inflammation in the subarachnoid space and that the outcome can be improved by modulation of the inflammatory response with dexamethasone. In central nervous system corticosteroid therapy decreases proinflammatory cytokine production (in monocytes, dendritic cells, astrogial cells and neutrophils) and increases the production of anti-inflammatory cytokines (6).

Since glucocorticoids act on multiple molecules of the TLR downstream signaling cascade, we could imagine the use of a specific therapy targeting TLRs (antagonist molecule, neutralizing antibodies) or their pathway (interleukin inhibitor: IL1, IL6, IL36; JAK inhibitor) would be effective to control AM.

Mechanisms of non-clearance of pneumococcal components within CSF over the time remain unclear. Pneumococci are extracellular pathogens that need to be phagocytosed to be eliminated. Their polysaccharide capsule protects them against phagocytosis, complement-mediated lysis and from T-cell and natural killer cell immunity. T-independent B lymphocytes that produce anti-polysaccharide antibodies are therefore the most important form of acquired immunity against these organisms. Antibodies interact with capsular polysaccharides and surface proteins and permit opsonisation with the help of activated complement. Thereafter phagocytose of pneumococci is favored. To explore the implication of a polysaccharide antibodies failure as a result of CPA persistence in CSF, we have conducted opsophagocytosis tests (performed with the patient's own bacterial strain) with specific antibodies against 24F polysaccharide in serum, as these tests are not validated in CSF, we consider that these tests reflect a systemic response effective in central nervous system. This test shows that these specific antibodies were present but not functional. Moreover, regarding the post-vaccine antibodies we also showed that they are present but ineffective. In 2013, Oishi et al. suggest that low opsonic activities may be explained by the lowed avidity of serotype specific IgG (7).

This lack of polysaccharide response could be explained in part by the physiological immaturity of T independent humoral response, in toddlers. A control after 5 years old will be proceed to eliminate an anti-polysaccharide immunodeficiency.

Nevertheless, we could hypothesize to explain chronic post-SP meningitis that adaptative immunity failure was “balanced” by an innate immunity response directed against a lifeless antigen. That is responsive of a chronic inflammatory response based on autoinflammatory pathway.

Further studies are needed to precise these potential interactions between innate and acquired immunity.

## Data Availability Statement

The original contributions presented in the study are included in the article/Supplementary Material, further inquiries can be directed to the corresponding author.

## Ethics Statement

Written informed consent was obtained from the legal guardian for the publication of any potentially identifiable images or data included in this article.

## Author Contributions

EJ: concept and design. ML, YD, KB, AB, and GB: aquisition of data. YD, FB, AB, KB, GB, ET, and SG: data analysis and interpretation. ML, EJ, and GS: drafting of manuscript and critical revision. All authors contributed to the article and approved the submitted manuscript.

## Conflict of Interest

The authors declare that the research was conducted in the absence of any commercial or financial relationships that could be construed as a potential conflict of interest.

## Publisher's Note

All claims expressed in this article are solely those of the authors and do not necessarily represent those of their affiliated organizations, or those of the publisher, the editors and the reviewers. Any product that may be evaluated in this article, or claim that may be made by its manufacturer, is not guaranteed or endorsed by the publisher.
