# Supplementary material for: Case Report: Persistency Pneumococcal Polysaccharide in Cerebrospinal Fluid During a Post Pneumococcal Chronic Aseptic Meningitis: Coincidental or (Auto-)Inflammatory Embers
Source: Front Pediatr. 2022 Feb 9;10:762457. doi: 10.3389/fped.2022.762457 (PMC8864159; doi:10.3389/fped.2022.762457)
Supplement: Supplementary file 1 [file Table_1.DOCX]

**Supplementary data 1**

Trio whole exome sequencing was performed for the proband and his unaffected parents. Sequences were aligned to human reference [GRCh37 - hg19](https://www.ncbi.nlm.nih.gov/assembly/2758) using BWA 0.7.17. Sequence variants were called using an in-house pipeline based on GATK HaplotypeCaller GATK 4.2.8.0 and DeepVariant 0.10 (<https://github.com/mobidic/MobiDL>) then merged and annotated with ANNOVAR with MPA prioritization algorithm (PMID:29689380). Common variants (minor allele frequency [MAF] >1.12% in GnomADv3) were filtered. Variants that were predicted to cause high or moderate impact in protein function and located in a gene list for Human Inborn Errors of Immunity based on the Classification from the International Union of Immunological Societies Expert Committee (PMID:31953710, 33598806) and patient-specific symptoms (HP:0001954; HP:0006946; HP:0000155; HP:0025439; HP:0002716) were prioritized. After this analysis, of 69 potential disease-causing variants, 27 were sequencing artifacts and of the others, we did not identify any sequence variation(s) that could explain the pathology.
